# Adaptations in Muscular Strength for Individuals With Multiple Sclerosis Following Robotic Rehabilitation: A Scoping Review

**DOI:** 10.3389/fresc.2022.882614

**Published:** 2022-05-06

**Authors:** Kailynn Mannella, Alan C. Cudlip, Michael W. R. Holmes

**Affiliations:** Department of Kinesiology, Brock University, St. Catharines, ON, Canada

**Keywords:** multiple sclerosis, rehabilitation, robotics, neurorehabilitation, strength

## Abstract

Muscular weakness and loss of motor function are common symptoms of multiple sclerosis. Robotic rehabilitation can improve sensorimotor function and motor control in this population. However, many studies using robotics for rehabilitation have overlooked changes in muscular strength, despite research demonstrating its utility in combating functional impairments. The purpose of this scoping review was to critically examine changes in muscular strength following robotic rehabilitation interventions for individuals with multiple sclerosis. A literature search of five databases was conducted and search terms included a combination of three primary terms: robotic rehabilitation/training, muscular strength, and multiple sclerosis. Thirty one articles were found, and following inclusion criteria, 5 remained for further investigation. Although muscular strength was not the primary targeted outcome of the training for any of the included articles, increases in muscular strength were present in most of the studies suggesting that robotic therapy with a resistive load can be an effective alternative to resistance training for increasing muscular strength. Outcome measures of isometric knee-extensor force (kg) (right: *p* < 0.05, left: *p* < 0.05), isometric knee flexion and extension torque (Nm) (*p* < 0.05), ankle dorsiflexion and plantarflexion torque (Nm) (all *p* < 0.05) and handgrip force (kg) (*p* < 0.05) all improved following a robotic training intervention. These adaptations occurred with sustained low resistive loads of hand grip or during gait training. This scoping review concludes that, despite a lack of studies focusing on strength, there is evidence robotics is a useful modality to improve muscular strength in combination with motor control and neuromotor improvements. A call for more studies to document changes in strength during robotic rehabilitation protocols is warranted.

## Introduction

Multiple sclerosis (MS) is a demyelinating and neurodegenerative disease of the central nervous system. It is estimated that every 5 min someone is diagnosed with MS globally (1). Despite the unpredictable and varying symptoms of the disease, one of the most clinically relevant and common complaints in this population is muscular weakness and loss of motor function (2). In most cases, rudimentary tasks such as bathing and feeding become increasingly more difficult. Approximately 45% of individuals with MS report loss of motor function within the first month of diagnosis and 90% report motor disability within the first year (3).

Muscular strength can be defined as the maximum voluntary force produced against an external resistance (4). Decreased motor function contributes to strength decrements in individuals with MS. In addition, those with MS often demonstrate muscle atrophy via denervation, leading to muscle morphological changes which can contribute to reduced muscular strength and overall functional impairments (5). Decreased force capability can lead to muscle weakness and affects motor or muscle performance (6). Robotic rehabilitation is a relatively novel rehabilitative tool for MS, but the literature demonstrates robotics can be effective for improving motor capabilities (7). Although there is a gap in the literature on the effects of strength training and functional outcomes, muscle weakness is a classic symptom of MS and there is evidence to suggest reductions in strength are associated with gait dysfunction, overall fatigue and increased disability (8). This knowledge leads to a hypothesis that reversing these effects with strength training may be important to improve gait and abilities of the upper limbs. Resistance training has been documented to improve overall muscular strength in MS by up to 11.6% following resistive exercise training 2-3 times per week for a range of 8-20 consecutive weeks (9). However, typical heavy load training or conventional resistance training may not be optimized or safely completed due to immobility and motor impairments of the disease.

Robotics can provide assist-as-needed algorithms, giving the user sufficient support to complete a task that may otherwise be unsuccessful. The high dose of repetitive movements applied with robotics show positive results for developing or restoring motor pathways, increasing muscular strength and improving function in MS (10). Neurologically, there are central motor impairments that contribute to muscle weakness (5) and repetitive, low resistance movements exploited with robotics, may contribute to neuromuscular adaptations and improvements in strength rather than increases in muscle size (hypertrophy) (11). In healthy populations, low resistance loads at a high dosage have been shown to elicit mean strength gains of 28% following resistance training programs, as compared to a 35.4% increase in mean strength following 1 repetition maximum training (12). These results support the indication that lighter loads can still elicit increases in muscular strength (12), which may be important for the MS population. Given the current literature, if increased muscular strength is the targeted outcome, low resistance loads with a high volume would be conducive to reduce the risk of injury and increase overall muscular strength. This is a novel topic that has yet to be exploited in rehabilitation interventions.

This research is important to the field because improvements in strength may provide the foundation for individuals to relearn functional tasks. The literature surrounding robotic rehabilitation for MS focuses primarily on motor control and performance measures, with improvements in muscular strength largely disregarded. Despite evidence that robotic devices are a beneficial and well-tolerated rehabilitative modality for inducing neuromuscular strength adaptations and that an increase in muscular performance can contribute to overall functional impairments (11), current systematic and scoping reviews have omitted to report on outcome measures surrounding grip force or muscular strength assessments (13, 14). Therefore, the purpose of this scoping review was to critically examine the literature to gain insight into adaptations in muscular strength following robotic rehabilitation interventions for individuals with MS. Interpretation of the findings will be critical within the broader context of MS because improved strength could mean delayed fatigue impairments, which could translate to task performance as well as optimal motor learning and rehabilitation.

## Methodology

This study was performed in accordance with the Preferred Reporting Items for Systematic reviews and Meta-Analyses (PRISMA) guidelines (15) and registered with PROSPERO, an international database of prospectively registered reviews (April 14, 2021; ID CRD42021246486).

### Search Strategy

Searches were conducted from six databases: MEDLINE, Ovid Healthstar, Cochrane Database of Systematic Reviews, Embase, Web of Science and CINAHL. The following search strings and keywords were used to carry out the search: multiple sclerosis OR (multiple sclerosis AND chronic progressive OR relapsing-remitting) AND robotic rehabilitation OR (robotics AND exercise therapy OR resistance training OR endurance training OR neurological rehabilitation) AND muscle strength OR (muscle contraction OR isometric contraction OR isotonic contraction). Search strategies were completed with the assistance of the institutional Librarian services and all database searches were completed on the same day (March 31, 2021).

### Eligibility Criteria

Eligibility criteria included peer-reviewed journal publications or conference proceedings that have been published in the last 20 years. Randomized controlled trials, controlled studies, cohort studies, case control studies, pilot studies and case series/case reports were included in the search. Studies were considered if they included participants with MS (any level/progression of disease) and used robotic rehabilitation (upper or lower extremity) for a minimum of 3 training sessions/days. Articles were excluded that did not directly measure muscular strength. For the purpose of this search, muscular strength was defined as a measurement of maximal voluntary isometric contraction (MVC) and the maximum force produced against an external resistance (4). Exclusion criteria comprised of books, editorials, dissertations, or if the article was not published in English. Studies were further excluded if the following were not included: outcomes (strength measures), intervention (robotic training), or based on study design (minimum of 3 training days).

### Methodological Approach

**Figure 1:**
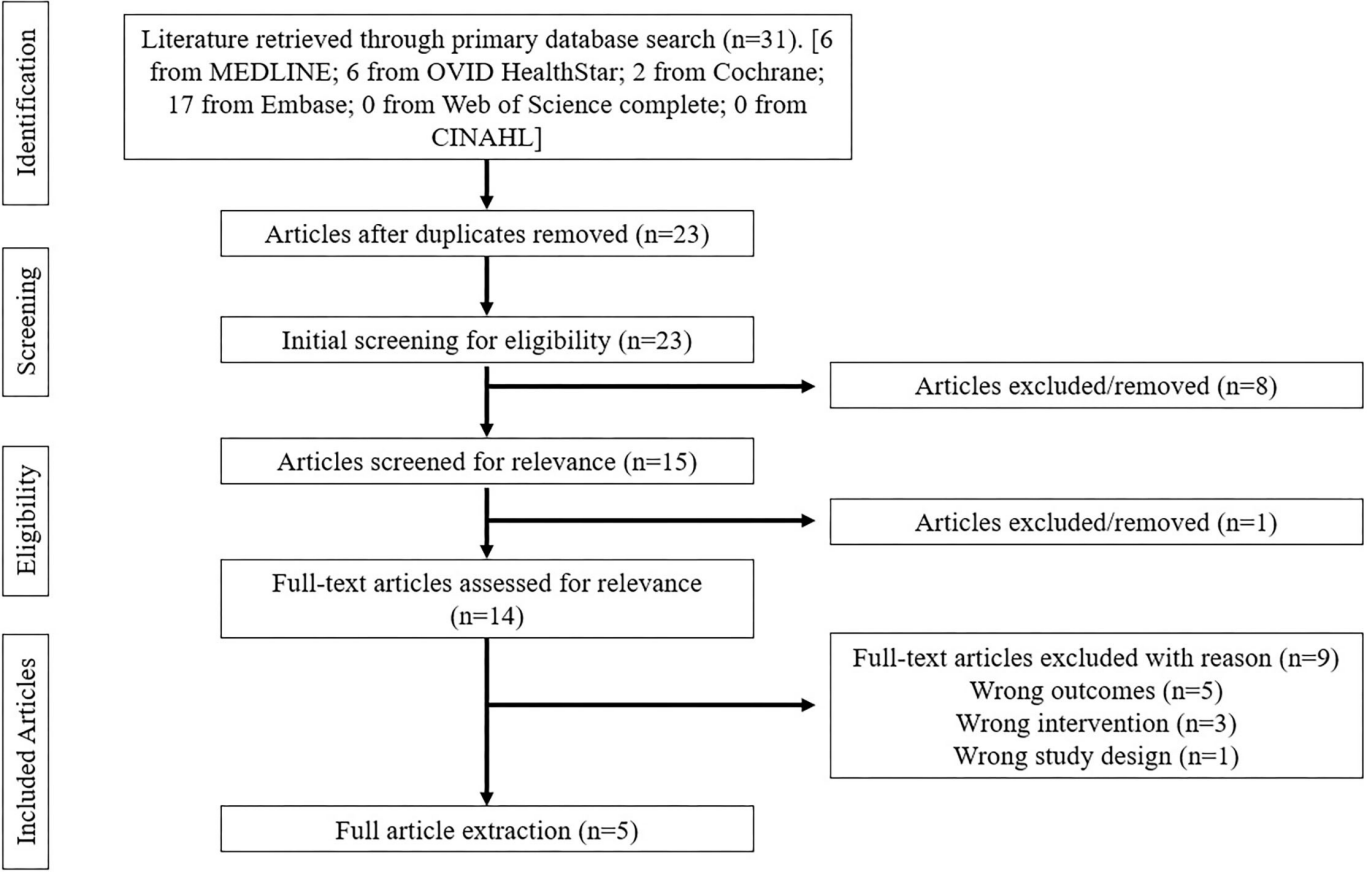
PRISMA flow-chart of literature search strategy and results.

All database searches were completed by a single researcher, followed by a double-blinded screening from two researchers. Articles that met the search terms within each database were extracted and collated into Covidence (Veritas Health Innovation, Melbourne, Australia). After removing duplicates, title/abstract screening was completed using the inclusion/exclusion criteria independently by two researchers. Following this initial screen, all qualifying articles underwent full-text screening using the same inclusion/exclusion criteria by the same researchers as the primary screening. Conflicts regarding inclusion/exclusion were resolved by a meeting between these two researchers until all disagreements were settled. A PRISMA flow diagram outlines the search strategy in Figure 1.

### Assessment of Methodological Quality and Risk of Bias

Methodological quality of the included studies was assessed with the Downs and Black Quality Checklist (16) and risk of bias assessment using Cochrane Handbook for Systematic Reviews of Interventions (17). The modifications to the original Downs and Black Quality Checklist included 16 items from the checklist that were relevant to this specific review (Table 1). Risk of bias assessment was based on bias arising from the randomization process, bias due to deviations from intended interventions, bias due to missing outcome data, bias in measurement of the outcome and bias in selection of the reported result (Table 2).

**Table 1:** Article quality assessment using Downs and Black checklist.

**Included Article**	**1**	**2**	**3**	**4**	**6**	**7**	**9**	**10**	**11**	**12**	**17**	**18**	**20**	**21**	**23**	**26**	**Total**
Beer et al. (18)	1	1	1	1	1	1	1	1	U	1	1	1	1	0	1	1	16
Feys et al. (19)	1	1	1	1	1	1	1	0	1	1	0	1	1	1	1	U	13
Lee et al. (20)	1	1	1	1	1	1	1	1	U	U	0	1	1	U	0	1	11
Lyp et al. (21)	1	1	1	1	1	1	1	1	U	U	1	1	1	U	U	1	12
Maris et al. (22)	1	1	1	1	1	1	0	1	U	0	1	1	1	U	0	1	11

*1, “yes” 0, “no”; U, “unable to determine”*.

**Table 2:** Risk of bias assessment using Cochrane Handbook for Systematic Reviews of Interventions.

**Included Article**	**Bias Domain**					
	**1**	**2**	**3**	**4**	**5**	**Overall**
Beer et al. (18)	L	L	L	L	L	L
Feys et al. (19)	L	L	L	L	L	L
Lee et al. (20)	L	L	L	L	L	L
Lyp et al. (21)	L	L	L	S	L	S
Maris et al. (22)	L	L	L	L	L	L

*L, “low risk”; H, “high risk”; S, “some concerns”*.

*Bias arising from the randomization process, (2) bias due to deviations from intended interventions, (3) bias due to missing outcome data, (4) bias in measurement of the outcome, (5) bias in selection of the reported result*.

## Results

### Search

The literature search resulted in a total of 31 articles: 6 (MEDLINE), 6 (Ovid Healthstar), 2 (Cochrane Database of Systematic Reviews), 17 (Embase). After duplications were removed and articles were screened for inclusion criteria, 5 articles were included for extraction and included pilot studies (*n* = 2), randomized controlled trials (*n* = 2) and controlled trials (*n* = 1). Data extraction included study subject size, duration of interventions, outcome measures of strength during baseline, post intervention and follow-up. Robotic training targeted the upper limb/reaching tasks (*n* = 2) and lower body/gait training (*n* = 3); upper limb focused training was unilateral training whereas the lower limb studies were primarily gait training interventions with bilateral exercises. Of the studies included, 3 reported MS phenotype which included participants with relapsing-remitting, primary progressive and secondary progressive MS, the remaining 2 studies did not report disease phenotype. Sample sizes ranged from 6 to 20 participants with a mean sample size of 12.7 ± 5.2. Expanded Disability Status Scale (EDSS) scores ranged from 6.0 to 8.0 and a disease duration ranging from 15 to 25 years. Number of training sessions ranged from 5 to 24 total active sessions with an average duration of 38.3 ± 11.0 minutes per session. Table 3 provides an overview of each study included in this review and Table 4 represents an article summary from the data extraction.

**Table 3:** Summary of extracted articles included in this re.

**Study**	**Aim of Study**		***n***	**EDSS**	**Disease duration (years)**	**Type of MS (RR/SP/PP)**
Beer et al. (18)	To evaluate feasibility and perform an explanatory analysis of the efficacy of robotic gait training in MS patients with severe walking disabilities.	Exp Con	14 15	6.0-7.5	15 ± 8	RR: 2 SP: 8 PP: 9
Lyp et al. (21)	To examine the effect of a robot-assisted body weight supported treadmill training on the walking ability of MS patients with impaired gait.	Exp Con	20 0	NR	NR	RR: 7 SP: 0 PP: 13
Lee et al. (20)	To perform and evaluate the efficacy of a 6-week robot-assisted training program for the treatment of ankle sensorimotor function in MS patients with lower limb impairments.	Exp Con	6 0	5.2 ± 2.5	16.0 ± 6.5	NR
Feys et al. (19)	To investigate the effects of additional robot-supported upper limb training in persons with MS compared to conventional treatment only.	Exp Con	17 0	8.0	25	NR
Maris et al. (22)	To investigate proof-of-concept efficacy of an individualized, robot-mediated training regime for people with MS and stroke patients.	Exp Con	13 0	6.5	17	RR: 5 SP: 6 PP: 2

**Table 4:** Detailed data extraction from included articles.

**Study**	**Robotic device**	**Days/ week**	**Session (n)**	**Session** **duration (min)**	**Strength measure**	**Baseline results** **(mean ±SD)**	**Post-intervention Results**	**Effect size (Cohen's D)**	**Follow-up results**
Beer et al. (18)	Lokomat	5	15	60	Knee-extensor strength— right/left (kg)	Right: 15.9 ± 7.5 Left: 13.6 ± 6.3	Right: 19.4 ± 7.5 Left: 16.9 ± 6.4	0.47 0.52	6-month: Results returned to baseline
Lyp et al. (21)	Lokomat	2	12	35	Flexion/extension muscles of the hip and knee joints (torque, Nm)	NR	Δ Torque 3.68 ± 1.64 – 7.06 ± 1.95	NR	No follow-up
Lee et al. (20)	Intellistretch	3	18	45	Dorsiflexion and plantarflexion MVC (Nm)	Dorsiflexion: 0.73 ± 12.72 Plantarflexion: 24.27 ± 9.89	Dorsiflexion: 12.08 ± 11.67 Plantarflexion: 34.97 ± 8.01	0.93 1.26	6-week: Dorsiflexion: 9.36 ± 11.69 Plantarflexion: 32.82 ± 0.49
Feys et al. (19)	Haptic Master	3	24	30	Handgrip strength (kg)	21.3 ± 12.0	21.0 ± 10.7	0.02	No follow-up
Maris et al. (22)	Haptic Master with I-TRAVLE	5	10	30	Handgrip strength (kg) Perceived strength (VAS)	Handgrip: 13.2 ± 9.1 VAS: 3.9 ± 3.0	Handgrip: 14.8 ± 1 VAS: 7.4 ± 5.1	0.15	3-month: Improved from baseline (p's = <0.05)

*Intervention volume, pre- post-intervention strength measurement results and effect size are reported. VAS; visual analog scale, NR; not reported*.

### Unilateral Upper Limb Robotic Training

The Haptic Master was used for robot-mediated upper limb rehabilitation training. In combination with I-TRAVL it allows for reaching tasks within a 3-dimensional workspace. Gravity compensation at the hand and elbow are individualized and allow for stabilized end-positions to aid the participant to a successful completion of the task. In both studies using the Haptic Master, participants underwent 30-minutes of task-oriented and dynamic movement training (10, 11). Specific outcomes and interventions of each study are reported in the article summary (Table 4). Handgrip force (kg) significantly increased in one of the two upper limb studies (*p* < 0.05), following a robotic training intervention between baseline and post training, and perceived strength (visual analog scale—*p* < 0.05) also significantly increased (22).

### Bilateral Lower Limb/Gait Robotic Training

The Lokomat system was used for the objective of robotic gait training. The Lokomat system was used as body weight supported orthosis and required extension and flexion of muscles of the hip and knee joints. This robotic rehabilitation also includes an interactive component inducing feedback-controlled training. In both studies using the Lokomat, leg movements were assisted by the robotic device with a preprogrammed physiological gait pattern. Body-weight support assistance was reduced as a progression throughout the training. Mean knee-extensor force increased by 3.5 and 3.3 kg for right and left side, respectively (right: *p* < 0.05 left: *p* < 0.05) (18); knee and hip flexion and extension torque increased by 7.1 and 3.7 Nm, respectively (*p* < 0.05) (21). The IntelliStretch was implemented to investigate ankle sensorimotor function in individuals with MS. The IntelliStretch is an ankle rehabilitative robot with controlled passive stretching and active movement. This robotic device also incorporates a computer game interface for feedback-control and participant engagement. The training program consisted of concentric and eccentric contraction of the dorsi and plantar flexors with a combination of passive and active stretching. Ankle dorsiflexion and plantarflexion torque increased by 11.4 and 10.7 Nm (all *p* = 0.028) (20).

## Discussion

The primary objective of this scoping literature review was to explore muscular strength adaptations following a robotic training program for a MS population, a novel topic in robotic rehabilitation research. The current literature appears to focus on the efficacy of robotics as a rehabilitation modality in respect to motor training and proprioception rather than strength outcomes, despite existing knowledge that muscular strength can be beneficial to one's quality of life (23). The overall purpose of improving strength is often to improve function, but adequate strength is fundamental for task success. Results of this review suggest that robotic rehabilitation can lead to strength adaptions following a robotic training program in both the upper and lower limbs. Regardless of the low resistance and a variety of dosage protocols in these studies, strength increases were still observed in this population. Despite a lack of studies focusing on strength included in this review, there is evidence robotics is a useful modality to improve muscular strength in combination with motor control and neuromotor improvements. A call for more studies to document changes in strength during robotic rehabilitation protocols and more care for effects on MS phenotype is warranted.

### Muscular Strength Adaptations

Although muscular strength was not the targeted outcome of the training in the included studies, increases in maximal contractions were present in most of the studies and illuminate the benefits of using robotics as a rehabilitation modality. Included studies with a focus on the upper limb showed increases in handgrip force through sustained end-effector manipulation. These muscular strength adaptations were supplementary to the goal of the intervention and occurred as a result of the training. Regardless of the mechanism behind the improvements in grip force, handgrip strength can be an important predictor and component of basic activities of daily living (22). For training interventions focused on the lower limb, voluntary eccentric muscle contraction of the quadriceps, and increased knee joint moment was required that, in turn, improved neuromuscular control and gait patterns, resulting in increased muscular strength compared to conventional gait-support training (18, 21).

None of the selected studies reported on training volume (i.e., number of repetitions, sets or training load) and only reported on the duration and frequency of the training sessions (Table 4). There is basis for improvement for future studies to report such information as training volume is critical when participating in conventional resistance training and is suggested to be of importance for rehabilitation as well (12). Due to low load requirements for MS patients, increased volume is necessary to increase strength. As such, the premise behind robotic therapy is to use a high volume of repetitions with minimal resistance added to the robotic device or acting against the participant's voluntary movements. It is plausible that the low loads exerted during gripping of the device or the eccentric movement of the quadriceps at a high dosage elicited adaptations in muscular strength in the studies included in this review. This theoretical speculation is based on evidence in healthy populations, proving that resistance training at low loads and a high dosage can still provoke strength gains (16). The work investigated here did not report changes in muscle size (hypertrophy), so it can only be speculated that the mechanisms of strength changes are from neural adaptations. This could be a deconditioning effect; all studies included were acute resistance trained (<12 weeks in duration) and likely, participants were not highly resistance trained prior to the intervention. With acute resistance training, there is often little to no change in muscle mass but still a greater max torque is produced (21). This is caused by neural adaptations as opposed to chronic resistance training whereby there is a larger increase in muscle mass and a smaller change in neural adaptations over time (24).

### Frequency of Interventions

In general, a consensus on proper therapy dosage and a link between total therapy time and strength outcomes is lacking in the MS literature. The studies included had therapy session frequencies ranging from 2 to 5 days, with total sessions completed ranging from 3 to 24 and duration times of 30–60 min. Typically, 8-week studies are chosen and are based on previous literature of non-robotic exercise focused studies (exercise/resistance training) (20, 23) and previous robotic literature (24, 25). However, only one study included in this review utilized an 8-week duration (19). All studies included in this review had time-dependent sessions as compared to repetition-dependent and omitted any resistance progressions throughout the therapy protocol despite knowledge that progression guidelines exist in the literature for resistance and cardiovascular exercise (6). Future work is necessary to develop guidelines for robotic therapy that are based on individual characteristics such as severity of disease, age, joint angle range of motion and progressions that clinicians can refer to like the physical activity guidelines that exist (26). If these general fitness principles are incorporated, the changes noted in strength following robotic rehabilitation may be even more apparent.

### MS Phenotype

There is clinical importance in individualizing rehabilitative treatment according to MS phenotype as symptoms, impairments and rehabilitative responses may differ between relapsing remittent, primary progressive and secondary progressive MS. Three of the five included studies (18, 21, 22) reported MS phenotype of their participants however, all neglected to categorize the results according to disease impairment. For example, it is unknown based on these findings if individuals with differing phenotypes or EDSS scores benefit from this rehabilitative modality. Beer et al. (18) included participants with severe walking impairments only (EDSS 6.0-7.5) and report improvements in knee-extensor strength post-intervention (Table 4). It is suggested that future studies consider MS phenotype as response to treatment can be affected by disease progression. It is important to note that no studies reported adverse effects to the training protocols. Thus, this therapy modality is well tolerated by individuals with MS.

### Upper Limb vs. Lower Limb

Robotics used in the included studies have examined clinical uses for both upper and lower limb rehabilitation. Lower limb robotic devices focused primarily on improving gait and mobility, and upper extremity robotics focused on tasks that require reaching and grasping, both are common movements affected by symptoms of MS and critical in everyday lives. In this review, three studies examined robotics for the lower limb and two studies examined robotics for the upper limb. One out of the two studies focusing on the upper limb did not show significant improvements in muscular strength and because of the lack of focus on handgrip force during the training, perceived fatigue and the inability to complete the task overpowered any potential improvements in strength outcomes (19, 22). Conversely, all studies of the lower limb reported improvements in muscular force and torque production following the intervention/training program. All studies with an upper or lower limb focus are limited by lack of progression overload and protocol individualization. Though, all studies showed significant improvements in most of their primary measures aside from muscular strength, including motor performance and accuracy.

### Limitations

There are a few limitations to this review that should be considered. Firstly, only five studies met the criteria for full-text analysis. Therefore, results are limited, and further work is necessary in this field. However, given the documented important link between muscle strength and daily function, this should highlight a call for more work in the area. Secondly, the included studies failed to report any rationale for the prescribed intervention including therapy dosage, frequency or load. This is a gap in robotic rehabilitative literature whereby intervention guidelines should be developed so clinicians know how long and how often they should prescribe robotic therapy to their patients. Lastly, none of the included studies considered MS phenotype or how response to treatment differs depending on level of disability or disease progression. There is not enough research in this field to yet identify this. For future work, this is necessary to investigate given that MS has a wide range of debilitating symptoms according to level of disability.

## Conclusion

The purpose of this scoping review was to critically examine changes in muscular strength following robotic rehabilitation interventions for individuals with MS. The current literature appears to have a focus on the effectiveness of robotic training comparative to conventional therapy, with strength outcomes acting as a secondary analysis. Targeted outcomes of the training were focused on motor performance but increases in muscular strength were present in 3 of the 5 studies and identifies robotics as a beneficial therapy tool for this population for a variety of outcomes. Included studies with a focus on the upper limb showed increases in handgrip force through sustained end-effector manipulation, while robotic interventions for the lower limb forced eccentric movement of the quadriceps, improving neuromuscular control and gait patterns. The premise behind robotic therapy is to utilize a high frequency dosage and this review indicates plausibility that the high repetitions with minimum resistance can stimulate improvements of handgrip force or torque of the quadriceps. Future work is vital to investigate progressions and individualization during a robotic rehabilitation training program to enhance the overall treatment. The low number of included studies in this review highlights the need for more studies to document changes in strength during robotic rehabilitation protocols.

## Author Contributions

KM searched all databases for possible articles, the article quality assessment using Downs and Black checklist and the risk of bias assessment using Cochrane Handbook for Systematic Reviews of Interventions. KM and AC performed the double-blinded screening of the search results. MH was a third party to resolve conflict/decision to include articles. All authors read and approved the final manuscript.

## Funding

MH is supported by the Canada Research Chairs program.

## Conflict of Interest

The authors declare that the research was conducted in the absence of any commercial or financial relationships that could be construed as a potential conflict of interest.

## Publisher's Note

All claims expressed in this article are solely those of the authors and do not necessarily represent those of their affiliated organizations, or those of the publisher, the editors and the reviewers. Any product that may be evaluated in this article, or claim that may be made by its manufacturer, is not guaranteed or endorsed by the publisher.
